# Digital Melting Curve Analysis for Multiplex Quantification of Nucleic Acids on Droplet Digital PCR

**DOI:** 10.3390/bios15010036

**Published:** 2025-01-10

**Authors:** Xiaoqing Dai, Meng Cao, Zunliang Wang

**Affiliations:** State Key Laboratory of Digital Medical Engineering, School of Biological Science and Medical Engineering, Southeast University, Si Pai Lou 2, Nanjing 210096, China

**Keywords:** multiplex detection, droplet digital PCR, digital melting curve analysis, fluorescence imaging, molecular diagnostics

## Abstract

We present a cost-effective and simple multiplex nucleic acid quantification method using droplet digital PCR (ddPCR) with digital melting curve analysis (MCA). This approach eliminates the need for complex fluorescent probe design, reducing both costs and dependence on fluorescence channels. We developed a convolutional neighborhood search algorithm to correct droplet displacement during heating, ensuring precise tracking and accurate extraction of melting curves. An experimental protocol for digital MCA on the ddPCR platform was established, enabling accurate quantification of six target pathogen genes using a single fluorescence channel, with an average accuracy of 85%. Our method overcomes the multiplexing limitations of ddPCR, facilitating its application in multi-target pathogen detection.

## 1. Introduction

Digital PCR has become increasingly important in nucleic acid quantification due to its high sensitivity and absolute quantification capability [1,2,3,4]. This technique partitions nucleic acid samples into tens of thousands of independent micro-reaction units, such as micro-wells or micro-droplets, enabling the sensitive detection of extremely low concentrations of target nucleic acids [5,6,7,8,9,10]. Multiplex detection is crucial in clinical diagnostics, particularly for infectious diseases, where co-infections involving multiple pathogens are common. Infections such as bloodstream infections or meningitis often involve multiple pathogens, making the ability to detect multiple targets simultaneously in a single reaction essential for accurately identifying all pathogens in a sample [11,12,13]. Performing multiplex detection in a single reaction also improves sample conservation, reduces costs by minimizing reagent and equipment usage, and simplifies the workflow, which is critical in resource-limited settings and high-throughput environments. Furthermore, a single reaction minimizes issues such as cross-contamination, inconsistent temperatures, and handling complexities that may arise when using multiple wells. Digital PCR offers the potential to precisely quantify multiple targets in a single test, improving diagnostic throughput, conserving sample volumes, and reducing costs, particularly in resource-limited settings. While digital PCR offers many advantages for quantitative nucleic acid analysis, multiplex digital quantification of multiple target sequences in a single amplification assay remains challenging. First, the number of fluorescent detection channels is physically limited, typically ranging from 1 to 6 channels [14,15,16]. Second, the necessity of designing specific probes for each target gene, as well as ensuring uniformity across different fluorescent detection channels, complicates multiplex detection, affects sensitivity after amplification, and increases detection costs [11,17]. Furthermore, as the number of target sequences increases, the challenges for multiplex digital PCR become increasingly significant [18,19].

Melting Curve Analysis (MCA) is typically performed after qPCR amplification to monitor DNA duplex denaturation during heating using fluorescent dyes (e.g., SYBR Green). The melting curve is generated by tracking fluorescence intensity changes with temperature, allowing for specific identification of amplified fragments based on their unique characteristics [20]. Fluorescent dyes-based MCA is a cost-effective method for multiplex nucleic acid detection, enabling differentiation of various targets by analyzing the melting characteristics of distinct gene fragments during heating, independent of the number of fluorescence channels. However, its current implementation in qPCR systems is primarily a qualitative technique and cannot be used for accurate quantification [21,22,23]. Digital MCA offers distinct advantages over traditional qPCR-based MCA, particularly its ability to perform multiplex detection and provide absolute quantification, capabilities that are more challenging to achieve accurately with qPCR. By analyzing the melting temperatures of various nucleic acid templates, digital MCA can effectively distinguish multiple target signals and facilitate multiplex quantitative analysis.

Currently, almost all reported research on digital MCA primarily relies on digital PCR platforms based on micro-well chips, where the fixed positions of the micro-wells enable simple extraction of melting curves from positive micro-wells [24,25,26,27,28]. However, these micro-well chips require photolithography or etching techniques to create tens of thousands of uniformly sized micro-wells on substrate materials like silicon wafers and quartz glass. The chip manufacturing process is both complex and costly, and minor errors in micro-well fabrication can directly affect the accuracy of nucleic acid quantification. The high costs of micro-well chip manufacturing have limited its broader use in digital MCA for multiplex nucleic acid assays [28]. Compared with micro-well chips, droplet-based microfluidic platforms offer superior flexibility by enabling the generation, control, and manipulation of discrete liquid volumes. These platforms facilitate precise handling of individual droplets for processes such as mixing, separation, analysis, and chemical reactions. The ability to generate large quantities of uniform droplets significantly enhances throughput, reliability, and cost-effectiveness, making them particularly useful in biomedical applications, including diagnostics, drug delivery, high-throughput screening, and digital PCR [29,30,31,32]. Digital PCR technology based on planar micro-droplet arrays provides significant advantages in operational flexibility and cost-effectiveness, demonstrating its greater potential for broader applicability [5,19,26,33].

In this study, we introduce a digital MCA method for multiplex nucleic acid quantification using a chip-free droplet digital PCR (ddPCR) platform based on interfacial vibration injection technology. However, when using this ddPCR platform, temperature variations can cause shifts in droplet position and shape, presenting challenges for melting curve extraction. So, accurately extracting the melting curve for each positive droplet necessitates precise correction of droplet positions during heating. In this study, we developed a convolutional neighborhood search algorithm to correct the displacement of positive droplets in each fluorescence image captured during the heating process. This method allows for accurate extraction of melting curves for target gene sequences within each positive droplet.

To evaluate multiplex detection performance, we selected six gene fragments targeting common respiratory bacterial pathogens—Staphylococcus aureus (cap5F), Escherichia coli (uidA), Klebsiella pneumoniae (yfkN), Acinetobacter baumannii (iucD), Haemophilus influenzae (atoE), and Streptococcus pneumoniae (lytA)—based on distinct melting temperatures calculated using the uMELT Quartz software (version 3.6.2) [34], enabling digital melt curve analysis (MCA) via the ddPCR system. The melting temperatures of each target fragment were determined using digital MCA and validated by high-resolution melting curve (HRM) analysis with real-time qPCR (RT-qPCR) instruments. Our experimental results demonstrated the effectiveness and feasibility of digital MCA for 2- to 6-plex nucleic acid quantification using a single fluorescence channel. The experimental results demonstrate that digital MCA, performed on a droplet array-based digital PCR platform, enables highly accurate multiplex molecular quantification.

## 2. Method and Materials

### 2.1. Experimental Instrument and Setup

In this study, the experiments of multiplex digital PCR based on melting curve analysis were performed using the Sniper DQ24 ddPCR system (Sniper Medical Technology Co., Ltd., Suzhou, China) for ddPCR and melting curve analysis. The SLAN-96P RT-qPCR instrument (Hongshi Medical Technology Co., Ltd., Shanghai, China) was utilized to conduct HRM analysis to validate the accuracy of our digital melting curve analysis. Sample separation was performed using the Sigma 3K15 desktop high-speed centrifuge (Sigma Laborzentrifugen GmbH, Osterode am Harz, Germany), followed by sample mixing and preparation with the MINI-12G simple centrifuge and QQXH-E oscillation mixer (Qiqian Electronic Technology Co., Ltd., Shanghai, China). Nucleic acid concentration was quantified using the Nano-300 microspectrophotometer (Aosheng Instrument Co., Ltd., Hangzhou, China).

### 2.2. Materials and Samples

For ddPCR experiments, the target amplification fragment was inserted into the pUC57 vector plasmid following artificial synthesis. The DNA sequence, along with upstream and downstream primers, was synthesized by Beijing Tsingke Biotech Co., Ltd. (Beijing, China). ddPCR consumables, including droplet generation oil, reagents, and kits, were provided by Sniper Medical Technology Co., Ltd. (Suzhou, China). Centrifuge tubes were obtained from Sangon Biotech Co., Ltd. (Shanghai, China). ROX reference dyes, which are used for signal normalization and compensation of fluorescence variations, were sourced from Shanghai BioScience Co., Ltd. (Shanghai, China). EvaGreen fluorescent dyes, which are utilized for real-time monitoring of the melting curve in each detected positive droplet, were obtained from Biotium, Inc. (Fremont, CA, USA). We chose EvaGreen, a saturated DNA dye, for melting curve analysis due to is high specificity, low background, and efficient binding to all minor grooves of double-stranded DNA, ensuring accurate melting profiles with minimal PCR interference [35,36,37]. The primer sequences are provided in Table S1.

### 2.3. Workflow of Multiplex ddPCR Detection Using Digital MCA

**Figure 1:**
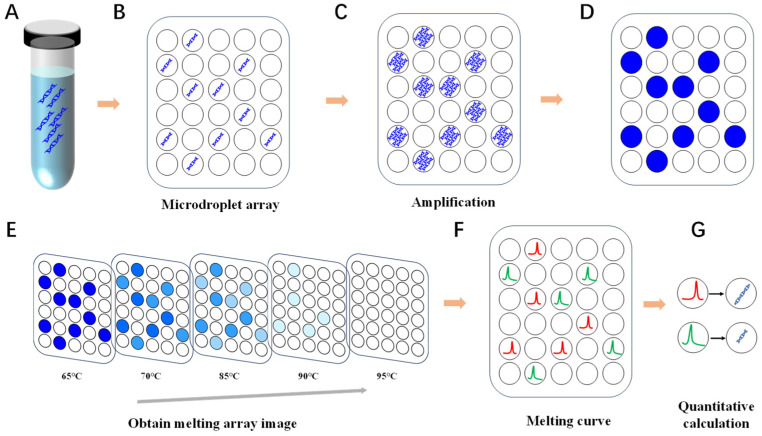
Schematic diagram of the digital PCR melting curve analysis, with details as follows: (**A**) A tube of nucleic acid sample is prepared for testing. (**B**) The sample is distributed into approximately 20,000 microdroplets of 0.8 nL, each using vibration injection technology on the ddPCR platform. (**C**) A total of 40 cycles of PCR amplification are performed for each droplet. (**D**) After the amplification is complete, the fluorescence images of the microdroplet array are captured. (**E**) A series of droplet array images are captured during heating. (**F**) The melting peak profiles of positive droplets can be obtained by taking the negative derivative of the melting curves, which are derived from the changes in the fluorescence signals from these droplets. (**G**) The positive droplets within the target’s Tm range are counted, which, along with the derived negative droplet count, allows for target concentration quantification using the Poisson distribution model.

As shown in Figure 1, the nucleic acid samples for ddPCR (Figure 1A) were uniformly partitioned into 20,000 microdroplets, each with a volume of 0.8 nL (Figure 1B). PCR amplification was then performed with 40 thermal cycles within each droplet (Figure 1C). After ddPCR amplification, an endpoint fluorescence image of the droplet array was captured, and the droplets were initially classified as positive or negative (Figure 1D). As the temperature increased, the fluorescence signals of positive droplets were tracked (Figure 1E), generating their melting peaks (Figure 1F). The number of positive droplets within the specific Tm range of each target’s melting peak was counted. Subtracting this from the total droplet count yielded the number of negative droplets for each target. The target concentration was then calculated using the Poisson distribution model based on the negative droplet count (Figure 1G).

**Figure 2:**
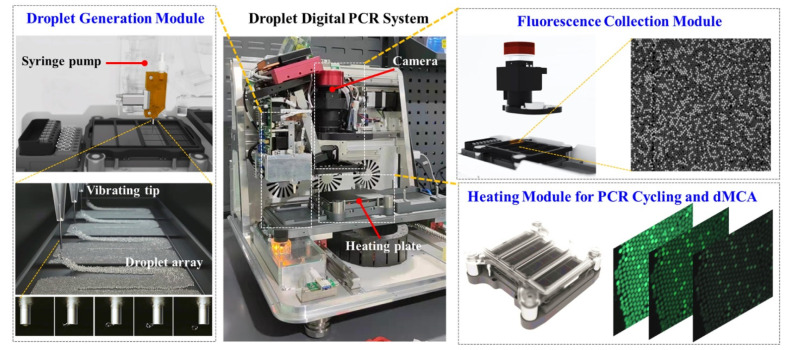
Structure and functional modules of the ddPCR platform used in this study. The platform is composed of three key modules: droplet generation, fluorescence collection, and heating. The droplet generation module employs an interfacial emulsification method driven by injection vibration technology to generate uniform water-in-oil droplet array. The fluorescence collection module captures the fluorescence signals from the amplified droplets, while the heating module regulates the temperature for PCR cycling and digital MCA.

Following the schematic of the digital MCA shown in Figure 1, Figure 2 illustrates the three key functional modules of the ddPCR system (Sniper DQ24) used in our study. The droplet generation module utilizes an interface emulsification method driven by injection vibration technology. In this process, the ddPCR reaction mixture (including nucleic acid samples) is injected into the oil phase using a syringe pump. The liquid sample containing the ddPCR reagents is emulsified into tens of thousands of uniform water-in-oil droplets as the vibrating tip induces shear forces at the oil-water interface, causing the two phases to mix. The droplet size can be controlled by adjusting the liquid flow rate and the vibration frequency of the tip. The fluorescence collection module captures fluorescence signals from the droplets after PCR amplification, enabling differentiation between positive and negative droplets. Additionally, the module monitors dynamic changes in fluorescence during the temperature ramp-up phase for melt curve analysis. The heating module provides precise thermal control for the ddPCR cycling and digital MCA. Together, these functional modules enable the integrated workflow for digital MCA.

### 2.4. Droplet Micro-Displacement Correction and Melting Curve Extraction

**Figure 3:**
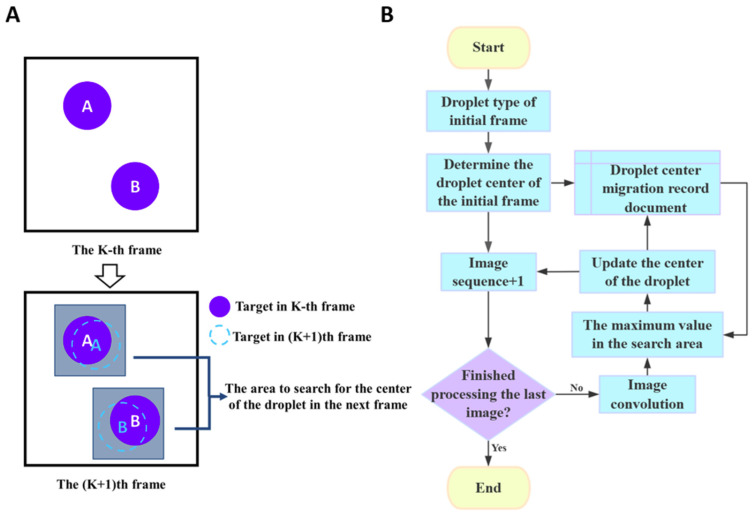
Schematic diagram (**A**) and flowchart (**B**) of the convolutional neighborhood search algorithm for droplet displacement correction.

After amplification, the droplet array undergoes further heating, leading to the micro-displacement of the droplets. To accurately extract the melting curves, it is essential to precisely identify and track each droplet during the heating process. Observing that the micro-displacement of nearly all droplets is actually smaller than their radius, we developed a convolutional neighborhood search algorithm to correct the center position of each displaced droplet (Figure 3). This algorithm can quickly track and locate each droplet at various temperatures, eliminating the redundant process of reidentifying and repositioning droplets in each frame of the fluorescence images.

To correct the droplet position, our algorithm utilizes a square convolution window of size Nr × Nr, where Nr corresponds to the average droplet radius in pixels. Each position in the convolution kernel is assigned a uniform weight of 1. Due to the gradient in gray values—decreasing from the center to the edge of each droplet—, the convolution operation consistently produces a regional maximum near the droplet center in the resulting matrix. As shown in Figure 3A, during the heating process, the displacement of droplets A and B between the k-th and (k + 1)-th frames typically does not exceed their radius. So, the droplet center from the previous frame is chosen as the seed point. The algorithm then searches for the maximum value within the Nr × Nr neighborhood around the seed point in the convolution result matrix. The index corresponding to this maximum value is then updated to represent the new center of the droplet. The flowchart of the convolutional neighborhood search algorithm is illustrated in Figure 3B.

The droplet melting curve illustrates the variation in fluorescence intensity during heating. Through the displacement correction, each droplet’s position at different temperatures can be accurately determined. So, the melting curve from each droplet can be derived by summing the image gray values, which represent the relative fluorescence intensity. The relative fluorescence unit (*RFU*) of a single droplet at a specific temperature is defined as follows:

(1) $$\mathrm{RFU}=h(x_{i},y_{i}),$$

where $\left(x_{i},{\;y}_{i}\right)$ is the coordinates of the droplet center, $h\left(x,y\right)$ represents the result of the convolution between the positive droplet image $I\left(x,y\right)$ and the square convolution kernel *G*. The convolution can be calculated as follows:

(2) $$h\left(x,y\right)=G*I\left(x,y\right),$$

where the square convolution kernel *G* has an odd side length of at least the droplet diameter, with weights of 1 assigned to the droplet region and 0 assigned to areas outside the droplet. After the droplet displacement correction, the fluorescence images collected at each temperature are used for the calculation of droplet *RFU* according to the resulting convolution matrix. Next, the variation curve of droplet *RFU* versus temperature is generated, and the droplet melting peak profile is derived from the negative first-order derivative of this original melting curve. The temperature at which the characteristic melting peak of the droplet melting curve occurs is defined as the melting temperature, determined using a peak detection algorithm. In melting curve analysis, as the temperature increases, the DNA duplex undergoes denaturation, transitioning into single strands, which results in a marked change in fluorescence. The most prominent fluorescence change occurs at the melting temperature (Tm). To accurately determine the Tm, the negative first-order derivative of the melting curve is commonly used. This approach highlights the rate of fluorescence change and identifies the point of maximum variation during denaturation. The use of the derivative helps overcome the challenge that the original melting curve may become too shallow at higher temperatures, making the Tm harder to pinpoint accurately. By analyzing the negative derivative of the curve, the melting peak is more clearly defined, enabling a precise determination of Tm.

### 2.5. Experimental Protocol Design for Digital MCA Using ddPCR

In single-target ddPCR experiments, the reaction mixture for each digital PCR amplification had a total volume of 22 μL, consisting of 11 μL of PCR Mix, 0.5 μL of 25 μmol/L ROX, 1 μL of 20× EvaGreen, 1 μL of forward primer (10 μmol/L), 1 μL of reverse primer (10 μmol/L), 3 μL of template, and 4.5 μL of aseptic, enzyme-free water.

**Figure 4:**
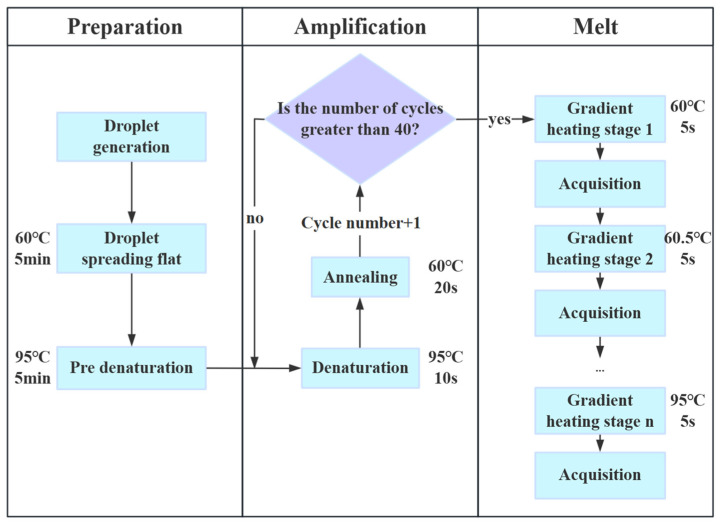
Protocol for digital MCA on the ddPCR platform.

The digital MCA protocol is illustrated in Figure 4. After generating the droplet array, it is heated at 60 °C for 5 min to ensure that the droplets are fully spread out and uniformly distributed before amplification. The PCR amplification begins with an initial denaturation at 95 °C for 5 min, during which the hot start polymerase is activated. PCR amplification is performed over 40 cycles, each consisting of 10 s of denaturation at 95 °C and 20 s of annealing/extension at 60 °C, taking approximately 40 min in total. Following amplification, the melting curve analysis begins by gradually heating the droplet array from 60 °C to 95 °C, with a 0.5 °C increment at each step. Accordingly, the fluorescence images of the droplet array are captured at every 0.5 °C, with a 5 s exposure time for each image. This phase takes approximately 30 min, followed by 10 min for data processing. In total, the entire process takes about 1 h and 35 min.

For the multiplex digital PCR assay, each reaction mixture has a final volume of 22 μL, comprising 11 μL PCR mix, 3 μL target DNA template at a specified concentration, 1 μL each of forward and reverse primers (10 μmol/L), 1 μL EvaGreen dye (20×), and 0.5 μL ROX reference dye (25 μmol/L). In 2- to 6-plex assays, the primer mixture contained 2 to 6 distinct primer sets.

## 3. Results and Discussion

### 3.1. Fluorescence Image Acquisition and Droplet Tracking

DNA melting curves are influenced by factors such as nucleotide sequence, guanine-cytosine (GC) content, amplicon length, and the composition of the PCR solution (e.g., salt concentration). These factors collectively determine the distinct melting profiles of different DNA sequences in digital MCA.

In our study, Tm values for the target genes were calculated using the uMELT Quartz software [34], which applies a sophisticated thermodynamic model that accounts for these multiple factors. The software predicts high-precision melting curves and Tm values by simulating the melting process without relying on a simple formula. In this work, we selected target gene fragments from six pathogenic bacteria associated with infectious diseases (cap5F, iucD, lytA, atoE, uidA, and yfkN) for multiplex detection analysis using digital MCA.

**Figure 5:**
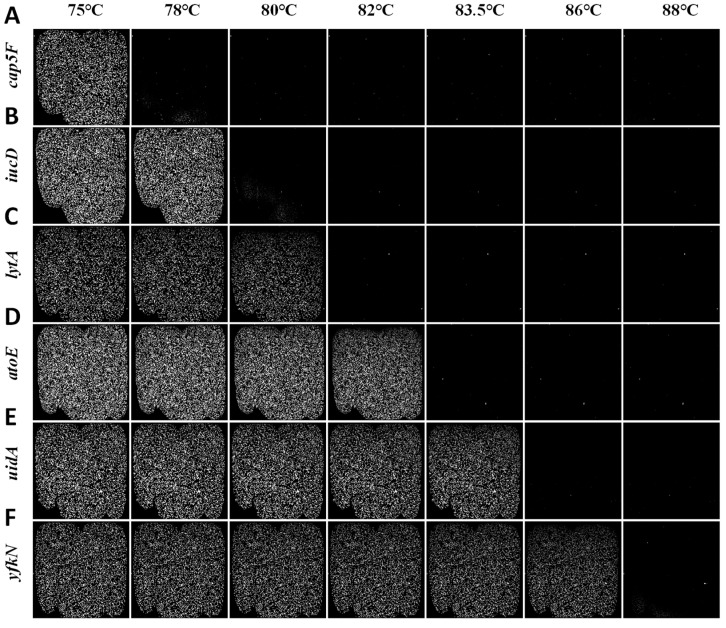
Fluorescence images of droplet arrays, each containing a single target gene fragment: (**A**) cap5F, (**B**) iucD, (**C**) lytA, (**D**) atoE, (**E**) uidA, and (**F**) yfkN, captured at different temperatures.

Figure 5 illustrates the image sequence of droplet arrays, each containing one of the six target genes, captured from a single fluorescence channel as the temperature increases. The first column shows the endpoint fluorescence images, captured after amplification for each target gene fragment, which are used to distinguish positive and negative droplets for ddPCR assays. The subsequent columns demonstrate the corresponding melting profiles, generated based on temperature-induced fluorescence changes.

Figure 5 shows that the fluorescence decay of the six target gene fragments exhibits different decay trends with increasing temperature. Melting temperatures are essential for capturing fluorescence image sequences in digital MCA, as they denote the specific temperatures at which nucleic acid fragments denature. Near these temperatures, fluorescence signals from droplet images experience significant changes. We first calculated the melting temperature (${Tm}_{cal}$) of six target gene individually by using the uMELT Quartz software. The predicted melting temperatures allow us to establish optimal temperature sampling windows, ensuring we capture critical fluctuations in fluorescence signals. Consequently, the fluorescence images of the droplet array were captured every 0.5 °C during the heating process from 65 °C to 92 °C.

**Figure 6:**
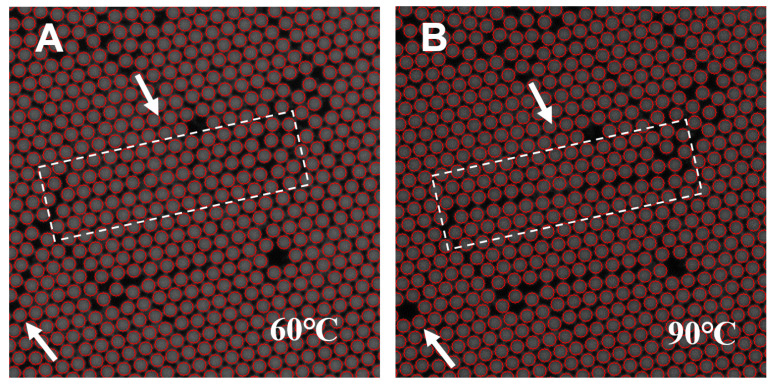
Droplet tracking through the micro-displacement correction method. The droplets (indicated by white arrows) at 60 °C (**A**) are accurately localized after minor displacement at 90 °C (**B**). Correctly identified droplets are highlighted by red circles.

By applying the convolutional neighborhood search method, the positions of droplets experiencing slight displacement due to heating were efficiently corrected in fluorescence images captured at different temperatures, enabling the extraction of target gene melting curves from each positive droplet. Figure 6 demonstrates the effectiveness of this micro-displacement correction for droplet tracking. As shown in the fluorescence images captured at 60 °C and 90 °C, some droplets (indicated by white arrows) exhibited slight displacement with increasing temperature but were still accurately tracked and identified (highlighted by red circles).

### 3.2. Calculation of Melting Temperatures via Digital MCA

**Figure 7:**
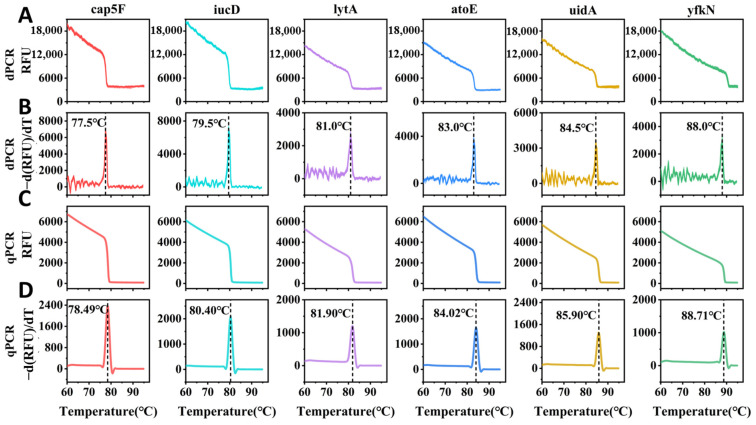
The original digital melting curve (**A**) after droplet digital PCR and its corresponding characteristic melting peak (**B**), derived from discrete differentiation of the original curve. The high-resolution melting curve (**C**) generated from RT-qPCR and its characteristic melting peak (**D**), calculated through discrete differentiation of the original high-resolution melting curve.

By accurately tracking droplets using the micro-displacement correction method, we successfully extracted the melting curves from the droplet image sequence, illustrating the fluorescence signal variation as the temperature increased (Figure 7A). From these melting curves, we further calculated the derivative to obtain the characteristic melting peak profile (Figure 7B), which allowed us to determine the melting temperatures (${Tm}_{ddPCR}$) for the six target fragments. To verify the accuracy of our digital MCA for specific target sequences, we performed high-resolution MCA at a temperature sampling rate of 0.013 °C on the same target samples using RT-qPCR. This generated both the melting curve (Figure 7C) and the corresponding melting peak profile (Figure 7D) for the precise determination of the melting temperature (${Tm}_{qPCR}$). Compared to the HRM curves (Figure 7C,D) obtained from the qPCR platform, droplet melting curves for target fragments exhibit minor noise but maintain a similar overall profile (Figure 7A,C), with nearly identical characteristic melting peaks (Figure 7B,D). This confirms the validity of our digital melting curve analysis.

The Tm values of cap5F, iucD, lytA, atoE, uidA, and yfkN, obtained from uMELT Quartz software (version 3.6.2), RT-qPCR, and ddPCR, respectively, are shown in Table 1 for comparison.

As illustrated in Table 1, the melting temperatures (${Tm}_{ddPCR}$) obtained from the digital melting curves are slightly lower than ${Tm}_{qPCR}$ derived from the HRM curves, with an average maximum difference of 1.1 °C. This slight Tm reduction can be attributed to two factors: the smaller, more dispersed reaction volumes in ddPCR, which improve heating efficiency, and the lower sampling frequency of digital melting curves, calculated using a forward-difference method that introduces minor sampling errors.

### 3.3. Experimental Validation of Multiplex Nucleic Acid Detection Using Digital MCA

To achieve multiplex nucleic acid quantification on the ddPCR platform using digital MCA, we designed fragment combinations for multiplex amplification based on the melting temperature distributions of six amplicons and their single-plex quantification accuracy, as illustrated in Table 2.

In this study, each melting curve corresponds to a specific droplet in the droplet array, with the melting temperature determined by the target molecules present in each droplet. After ddPCR amplification, fluorescence images of the droplet array are captured to classify each droplet as positive or negative based on fluorescence intensity. Positive droplets indicate the presence of at least one amplified target sequence, while negative droplets exhibit little or no fluorescence. The droplets are then subjected to melting curve analysis. As the temperature increases, each droplet’s fluorescence signal is monitored, revealing its unique melting profile. This profile corresponds to the specific Tm range of each target’s melting peak, which is distinct for each target. The number of positive droplets whose melting profiles match the Tm range of a given target is counted, and the number of negative droplets is determined by subtracting the positive droplets from the total droplet count. This step ensures that droplets are categorized based on their specific Tm range, rather than the intensity or height of the melting peaks. Finally, the concentration of each target is calculated using the Poisson distribution model, based on the count of negative droplets. In this approach, melting curve analysis is crucial for target differentiation and accurate droplet classification, which forms the basis for multiplex digital quantification.

**Figure 8:**
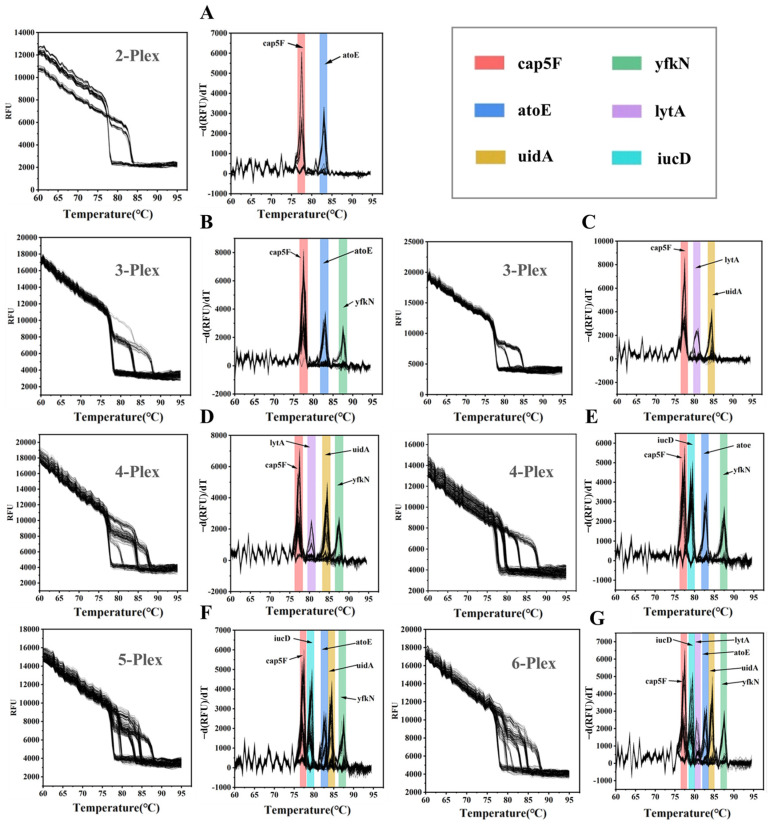
Digital melting curves (**A**–**G**: **left**) and corresponding melting peak profiles (**A**–**G**: **right**) of nucleic acid fragments within all positive droplets for 2- to 6-plex ddPCR assays. Different colored bars indicate the melting peaks for six target nucleic acid sequences: red for cap5F, blue for atoE, yellow for uidA, green for yfkN, purple for lytA, and cyan for iucD.

Figure 8A–G illustrates the original melting curve along with the corresponding melting peak profiles for 2- to 6-plex ddPCR detection. As shown in Figure 8A, for the 2-plex detection, droplets containing only cap5F target fragments show a melting peak at 77 °C, while droplets containing only atoE target molecules exhibit a melting peak at 82.5 °C. Importantly, droplets containing both target molecules display two distinct melting peaks, each corresponding to one of the target sequences. This clear distinction between melting peaks allows us to identify and quantify each target within the same droplet, a feature that distinguishes our approach from conventional fluorescence-based multiplex PCR methods, which are prone to fluorescence cross-talk and channel interference. For the 3- to 6-plex detection (Figure 8B–G), we observe similar results, with each target displaying a unique melting peak within the melting curve. Notably, even as the number of targets increases, melting temperature separation remains clear, allowing for the accurate identification and quantification of each target even at higher multiplex levels. This is typically not achievable with traditional methods, where increasing the target number often leads to overlapping signals and reduced quantification accuracy. By using a single fluorescence channel for dye-based melting curve analysis, our method enables multiplex detection without relying solely on the number of fluorescence channels, thus reducing costs and simplifying the experimental setup. This makes digital MCA particularly suited for high-throughput applications and point-of-care diagnostics, where minimizing both complexity and cost is essential.

### 3.4. Quantitative Accuracy of Multiplex Nucleic Acid Detection

**Figure 9:**
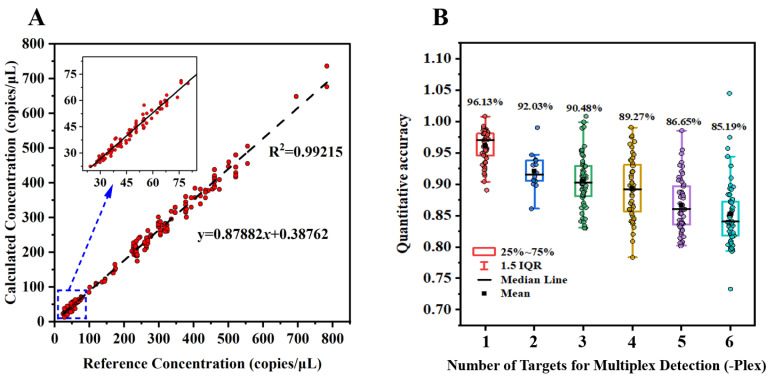
Quantitative accuracy of multiplex nucleic acid detection based on digital MCA: (**A**) Linear correlation between target gene concentrations calculated from multiplex quantification and reference template concentrations. (**B**) Relationship between multiplex quantitative accuracy and the number of target genes.

In Figure 9A, we show the regression analysis between the calculated concentration ($C_{cal}$) from the digital MCA in 2- to 6-plex ddPCR tests, and the corresponding reference template concentration ($C_{ref}$). The strong linear correlation (R^2^ > 0.98) between these two variables confirms the reliability and accuracy of our digital MCA method. This strong correlation indicates that the digital MCA method can provide accurate and reproducible nucleic acid quantification, even in complex multiplex assays with up to 6 targets. This result shows a significant advantage over conventional multiplex qPCR methods, as digital melt curve analysis not only enables the simultaneous detection of multiple targets but also allows for high-precision absolute quantification of each target. Meanwhile, relative quantification accuracy using the digital MCA method is determined by the ratio of $C_{cal}$ to $C_{ref}$. In Figure 9B, we examine the relationship between multiplexing levels and quantification accuracy. As expected, accuracy declines with an increase in the number of targets, with an average accuracy of 92% in 2-plex detection, decreasing to approximately 85% in 6-plex detection. This decrease is attributable to several factors inherent to multiplex ddPCR experiments, including primer compatibility, competitive binding within the droplets, and nonspecific amplification [38]. As the number of primers increases in a droplet, nonspecific interactions rise, leading to reduced amplification efficiency for each individual target. Consequently, some droplets may contain insufficiently amplified target fragments, resulting in false negatives during melting curve analysis. These issues, however, are not unique to our method and are common challenges in multiplex PCR assays. Despite these challenges, our method performs exceptionally well, maintaining 85% quantification accuracy even in 6-plex detection.

As demonstrated in Figure 8 and Figure 9, by establishing a reliable digital melting curve analysis (MCA) method on the planar droplet ddPCR platform, we enable multiplex detection using a single fluorescence channel and fluorescent dyes. This approach addresses key limitations of traditional ddPCR, including reliance on multiple fluorescence channels, complex probe design, and the associated costs and risks of signal cross-talk. The simplicity of fluorescent dyes makes our method not only more cost-effective but also scalable, especially in resource-limited environments, thereby overcoming the traditional limitations of ddPCR in multiplex applications.

In our ddPCR system, limited temperature control precision (0.5 °C) poses a challenge for digital MCA. When the melting temperature differences between target genes are smaller than 0.5 °C, distinguishing their melting peaks becomes difficult. To address this issue, enhancing temperature control precision or using higher-resolution sampling can improve peak differentiation. Additionally, selecting target genes with greater melting temperature differences can reduce peak overlap and improve analysis accuracy.

In this work, the dye-based melting curve analysis method is simple and cost-effective. However, dye-based fluorescence detection is prone to interference from primer dimers, which can reduce the accuracy. Therefore, optimizing primer design to minimize dimer formation is essential for enhancing the accuracy and specificity of multiplex detection. Finally, integrating advanced data analysis algorithms and machine learning tools will facilitate more effective interpretation of melting curve data, ultimately increasing the sensitivity and accuracy of multiplex detection. These strategies will enable better identification and differentiation of similar melting curves, thereby improving overall detection performance.

While multiplex detection with non-specific dsDNA binding dyes is feasible in qPCR, it is primarily qualitative and lacks precise quantification, especially in complex samples. In contrast, our ddPCR-based digital MCA approach enables both accurate detection and quantification of multiple targets within a single reaction. This makes it particularly effective for detecting and quantifying multiple pathogens, including co-infections, providing a powerful tool for infectious disease diagnostics.

This study successfully demonstrates multiplex quantitative detection of six targets using a single fluorescence channel on the ddPCR platform, highlighting the feasibility of multiplexing with limited fluorescence channels. Building on this approach, integrating additional fluorescence channels offers the potential for ultra-high multiplex detection in the future. However, challenges such as optimizing channel integration and managing fluorescence interference at higher multiplex levels must be addressed to ensure accuracy. These advancements could enhance diagnostic capabilities, enabling broader applications in molecular diagnostics and clinical testing.

## 4. Conclusions

In this study, we presented a fluorescent dye-based digital melting curve analysis (MCA) approach for multiplex detection using a ddPCR platform. We developed a convolutional neighborhood search algorithm to correct droplet position shifts during heating, enabling precise extraction of melting curves from positive droplets. To reliably capture fluorescence variation during heating, we designed a digital MCA protocol that integrates temperature control and fluorescence imaging of the droplet array. We validated this method by simultaneously detecting six target fragments from six common pathogens using a single fluorescence detection channel on the ddPCR platform, achieving an average accuracy of 85%. The melting temperatures (Tm) of each target fragment were determined through digital MCA and further confirmed by HRM with RT-qPCR instruments. In conclusion, we introduced a novel method for multiplex quantitative detection of six targets using a single fluorescence channel on the ddPCR platform. This approach addresses current multiplexing limitations and offers a cost-effective, accurate solution for nucleic acid quantification. The integration of additional fluorescence channels in the future could further enhance multiplexing capabilities, enabling ultra-high multiplex detection. These advancements have significant potential to advance molecular diagnostics and expand the clinical applications of ddPCR.

## Figures and Tables

**Table 1:** Comparison of the melting temperature *Tm* by calculation, qPCR and ddPCR of different target fragment.

Target Fragment	Amplicon Length (bp)	${Tm}_{cal}$ (°C)	${Tm}_{qPCR}$ (°C)	${Tm}_{ddPCR}$ (°C)
cap5F	175	77.75	78.52 ± 0.05	77.25 ± 0.65
iucD	193	79.50	80.37 ± 0.10	79.00 ± 0.50
lytA	110	81.75	81.83 ± 0.03	80.50 ± 0.50
atoE	168	84.00	83.97 ± 0.08	82.75 ± 0.65
uidA	215	86.00	85.68 ± 0.11	84.50 ± 0.50
yfkN	223	90.00	88.77 ± 0.11	87.25 ± 0.65

**Table 2:** Designed combinations of target fragments for multiplex detection using digital MCA.

Target	cap5F	iucD	lytA	atoE	uidA	yfkN
2-plex	+	−	−	+	−	−
3-plex	+	−	−	+	−	+
3-plex	+	−	+	−	+	−
4-plex	+	−	+	−	+	+
4-plex	+	+	−	+	−	+
5-plex	+	+	−	+	+	+
6-plex	+	+	+	+	+	+

Note: “+” denotes the presence of specific target fragments and “−” indicates their absence.

## Data Availability

The data supporting the findings of this study are available within the article and its Supplementary Materials. Additional data can be obtained from the corresponding author upon reasonable request.

## Supplementary Materials

The following supporting information can be downloaded at https://www.mdpi.com/article/10.3390/bios15010036/s1: Table S1: Primer sequence used for multiplex nucleic acid detection using digital MCA.
