# Supplementary material for: Digital Melting Curve Analysis for Multiplex Quantification of Nucleic Acids on Droplet Digital PCR
Source: Biosensors (Basel). 2025 Jan 10;15(1):36. doi: 10.3390/bios15010036 (PMC11764372; doi:10.3390/bios15010036)
Supplement: Supplementary file 1 [file biosensors-15-00036-s001.zip › biosensors-3352472-supplementary.pdf]

Supplementary Material

Digital Melting Curve Analysis for Multiplex Quantification of Nucleic Acids on Droplet Digital PCR

Xiaoqing Dai[#], Meng Cao[#], Zunliang Wang*

State Key Laboratory of Digital Medical Engineering, School of Biological Science and Medical Engineering, Southeast University, Si Pai Lou 2, Nanjing 210096, China.

[#] **These authors contributed equally to this work.**

***Correspondence and requests for materials** should be addressed to E-mails: zlwang@seu.edu.cn (Zunliang Wang)

SUPPLEMENTAL TABLES

Table S1. Primer sequence used for multiplex nucleic acid detection using digital MCA

**Target gene**	**Primer sequence（5' to 3'）**	**Fragment length（bp）**
cap5F	Forward (AGTCACGTCTCGATCGAACA)<br>Reverse (GAAACTTGACCACGATCCGG)	175
iucD	Forward (GGCTGGACATCATCAACTGC)<br>Reverse (GTCGGCCTGATCTCGTATGA)	193
lytA	Forward (GCACACTCAACTGGGAATCC)<br>Reverse (ATGCAACCGTTCCCAACAAT)	110
atoE	Forward (CTGGTGTTGCGGCTAAAAGT)<br>Reverse (TCATTAACTGGGGCTTCGGT)	168
uidA	Forward (CGACTGGGCAGATGAACATG)<br>Reverse (TACTCCACATCACCACGCTT)	215
yfkN	Forward (TACACAATCGCCCGTTGAAC)<br>Reverse (CCCGGTTAGATCCATGGTGA)	223
